# School assets and bullying in Chinese youth: A multiple mediation model of intentional self-regulation and internet gaming disorder

**DOI:** 10.3389/fped.2022.947869

**Published:** 2022-07-28

**Authors:** Xiong Gan, Ke-Nan Qin, Guo-Xing Xiang, Xin Jin, Cong-Shu Zhu

**Affiliations:** ^1^Department of Psychology, College of Education and Sports Sciences, Yangtze University, Jingzhou, China; ^2^Department of Psychology, College of Education and Sports Sciences, Yangtze University College of Technology and Engineering, Jingzhou, China

**Keywords:** school assets, bullying, intentional self-regulation, internet gaming disorder, adolescent

## Abstract

Bullying is a severe social problem affecting young people all over the world. Previous studies suggested that engagement in bullying had massive effects on teenagers’ physical and psychological development. It is critical and necessary to investigate the antecedents and underlying mechanisms of this phenomenon among young generations. The present study, based on the positive youth development perspective and the developmental assets theory, attempts to explore the positive factors in the school subsystem that could effectively prevent adolescents from bullying, as well as the multiple mediation effects of intentional self-regulation (ISR) and internet gaming disorder (IGD). In this study, we adopted a two-wave design and recruited a sample of 768 Chinese adolescents using a randomized cluster sampling method in the post-pandemic era. The results revealed that T1 school assets significantly and negatively predicted T2 adolescent bullying. Furthermore, T2 ISR and T2 IGD mediated the association between T1 school assets and T2 bullying separately and sequentially. Overall, school resources play a protective role in adolescent development and could effectively prevent them from negative outcomes. These current findings contribute to the literature by providing a further understanding of the direct and indirect protective effects of school assets on adolescent bullying. Moreover, practitioners could also benefit from these findings in preventing and intervening in bullying in the school subsystem.

## Introduction

Bullying is a widespread and complicated global public health issue (1, 2), which is defined as a sort of aggression that has three main characteristics: intentional injury; repeated behavior over a period of time; and an imbalance of power between the parties (3). Bullying is prevalent among young generations worldwide. For instance, researchers have reported that the rates of bullying (including bullies, victims, and bully victims) vary from 15.9 to 40.0% across Asian countries (4–9). In China, more than one fifth of children and teenagers experienced involvement in bullying at school (10, 11). Abundant studies have shown that Chinese adolescents who engage in bullying behavior exhibit lower life satisfaction and subjective well-being (12, 13) and also experience mental health problems (14), such as depression and anxiety (15), which can even lead to suicide (16). Furthermore, cross-cultural evidence suggests that bullying will cause serious psychosomatic problems in children and adolescents (17), including personality disorders, psychotic symptoms, and poor school safety (18–20). Bullying also resulted in a variety of negative externalizing outcomes that hampered their adaptive development, such as low emotional adjustment, academic difficulties, substance abuse, self-harm, weapon possession, violent behavior, and delinquency (21–25). It is clear that bullying can jeopardize school safety in elementary and secondary schools and the harmony and stability of society as a whole. As a consequence, it is necessary to look into the preventive and protective factors regarding bullying.

Given the high prevalence and negative effects of bullying, previous research has revealed some risk factors (e.g., violence exposure, deviant peer affiliation) for bullying and aggression to formulate and evaluate intervention projects (26, 27). However, this “problem prevention” focused view actually neglects the development of students’ strengths and potential (28). Fortunately, positive psychology has emerged as a new avenue for better understanding youth development in recent years, which is the positive youth development (PYD) perspective. The PYD highlights that parents, schools, and communities should provide more developmental opportunities and assets for youth to promote healthy growth and prevent negative outcomes (29). From this emerging perspective, this study aims to explore the positive factors from school that could prevent adolescents from bullying in the Chinese context.

### School assets and adolescent bullying

As they enter secondary school, Chinese teenagers dedicate more energy and time to school than their counterparts in Western countries (27). At this time, the focus of adolescent socialization gradually shifts from parent-child relationships to teacher-student and peer relationships (30). Bullying behavior often occurs with the development of peer relationships. As such, the search for factors that influence bullying is predominantly focused at the school level (31). Researchers found that favorable school factors contributed to improving well-being and reducing aggression (32, 33). According to social-ecological systems theory (34), individual development is influenced by a combination of different social systems, such as family, school, and community. As prior research has concentrated more on the risk factors for bullying (26, 27), this study will emphasize the positive factors in the school system.

School assets, which are derived from the developmental assets framework, are a broad term that encompasses protective factors in the school context (35). The PYD perspective advocates that juveniles exposed to high levels of environmental assets are less likely to engage in dangerous conduct and are more likely to develop in a healthy manner (36). A considerable number of cross-sectional and longitudinal studies have pointed out that the warmer school climate is a critical factor in keeping students away from aggression and bullying (37–39). When students had a positive perception of their school environment, they typically expressed a lower frequency of peer victimization and assault (40–42). The prior literature indicated that teacher-student relationships as a developmental asset from school was helpful in reducing the rise of teenage bullying (43–45). Conversely, conflictual teacher–student relationships exhibited significantly adverse effects on both bullying and victimization (46). Meanwhile, positive school belonging and school connectedness have played significant roles in avoiding bullying and aggressive behavior among adolescents (47–49). The above research evidence and theories imply that adequate school assets will benefit adolescents in decreasing bullying. Given the lack of previous research of school assets on bullying, this study proposes that school assets will negatively predict adolescent bullying (Hypothesis 1).

### The mediating role of intentional self-regulation

The triadic codetermination theory emphasizes that human functioning is a dynamic interaction between the individual’s personality traits, their behaviors, and the social environment (50). In addition to the influence of bullying from the school system, the individual’s personality traits may play a mediating role in this relationship. Intentional self-regulation (ISR) is a series of actions in which people purposefully coordinate their needs, assets, and personal aspirations to attain their developmental goals by using a variety of methods (51). During puberty, this ability to self-regulate matures and begins to play a major role (52). According to the relational-developmental-systems model, ISR can help individuals better manage themselves and achieve positive interactions with their surroundings, thereby reducing problem behavior (53).

To begin with, several empirical studies found that positive environmental assets in schools benefit the development of ISR for young people in China (54, 55). For instance, Lin et al. (54) have demonstrated that high school students well connected to their schools have access to more external assets, thus facilitating the enhancement of ISR. Besides, Chang and her colleagues conducted a longitudinal study on 1051 Chinese adolescents and revealed that school assets had a significant and predictive effect on subsequent ISR (32).

Second, youth with high levels of ISR not only developed positively, but they also experienced a few negative adjustment problems (56). On the one hand, ISR is beneficial for increasing adolescents’ well-being and for developing better future orientation psychological attributes (32, 54). Moreover, a longitudinal study about ISR and pro-social behavior showed a bidirectional relationship between ISR and pro-social behavior toward strangers during early adolescence (i.e., from age 12 to 14) (57). On the other hand, ISR is an important protective factor against risky or problematic behaviors among adolescents (55, 58, 59). Wang et al. (60) investigated 1406 adolescents in China and indicated that higher ISR would reduce youth engagement in aggressive behavior. Therefore, we speculate that the ISR may predict fewer occurrences of bullying. Through reviewing theories and literature on the associations between school resources, bullying and ISR, this study hypothesizes that ISR will mediate the relationship between school assets and bullying among Chinese adolescents (Hypothesis 2).

### The mediating role of internet gaming disorder

Once an individual is involved in a problem behavior, it is of higher risk for him or her to develop more types of problem behaviors (61). This provides a new approach for explaining bullying behavior and other problem behaviors such as internet gaming disorder (IGD). IGD refers to an individual’s uncontrollable, excessive, and compulsive use of online games that causes social and/or emotional problems (62). Young people in Asian countries were found to have higher rates of IGD (63), which varied from 5.4 to 17.7% (64–68). In China, the prevalence rate of IGD can reach 17.0% (64). Similar to bullying, IGD will also lead to a series of physical, mental, and behavioral problems (69–71).

According to social control theory (72), a favorable school environment helps students develop “bonds” and social norms that limit their offending behavior, such as the IGD. Xiang et al. (73) found that developmental assets, incorporating school assets, negatively predicted adolescent IGD through a longitudinal study. In a study of 500 Chinese students ranging from 12 to 17 years old, Yu et al. (74) showed that school climate could alleviate their problematic online game use. In addition, poor school engagement also predicts school dropout and other problematic behaviors among youth (75). Specifically, Tian et al. (30) and Salmela-Aro et al. (76) found that levels of school engagement were a significant negative predictor of both excessive internet use and IGD. Besides, Li et al. (77) also revealed that school connectivity can be effective in preventing problematic online game use. Therefore, it is reasonable to assume that quality school assets will reduce IGD among teenagers.

Furthermore, Karatoprak et al. (78) noted that internet addiction was predictive of both bullying and victimization. An investigation also revealed the predictive effect of internet addiction on bullying (79). Likewise, Kim et al. (80) found that online game addiction was a risk factor for school violence perpetration. These findings suggest that IGD may also be closely related to bullying. In accordance with the general learning model (GLM) (81), any external stimulus that individuals receive will have an effect on themselves through some learning mechanism. So, if an individual indulges in IGD for a prolonged period of time, then it can alter the individual’s internal assessment and decision-making functions, which can lead to bullying. Reviewing the complex associations between school resources, bullying, and IGD, we speculate that IGD may play a mediating role in the association between school assets and bullying (Hypothesis 3).

### Intentional self-regulation and internet gaming disorder

Adolescents able to develop positively can contribute more to society and are less likely to exhibit internalizing psychological issues and externalizing risk behaviors (82). These contributions covered a wide range of actions taken to promote their own, their family, community, and even societal well-being (83). ISR is an important ability and has a prominent place in adolescent health development (84, 85). The Selection, Optimization, and Compensation (SOC) model (86) states that individuals will better manage the relationship between themselves and their environment through selection, optimization, and compensation. This model underpins the concept of ISR. Extensive longitudinal surveys have demonstrated that ISR can positively predict well-being maintained across years and can also predict negative developmental indicators of adolescents, including depression, substance abuse, delinquent behavior, and other risky behaviors (87–89). Further, it has also been shown that individuals with high levels of ISR appear to have lower levels of social networking site addiction and problematic online game use (74, 90). So, ISR may also be a predictor of IGD. Considering the above empirical evidence and theory, this study hypothesizes that ISR and IGD will serially mediate the relationship between school assets and adolescent bullying (Hypothesis 4).

### The present study

**FIGURE 1:**
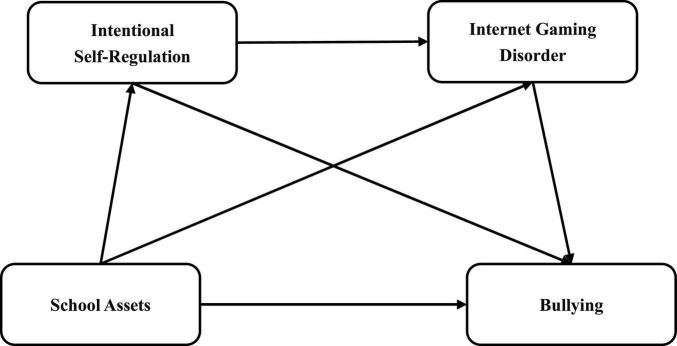
The conceptual multiple mediation model.

For a long time, studies on problem behavior have often centered on ‘fixing deficits’, but very few studies have investigated the relationship between school assets and adolescent bullying from the perspective of PYD. In light of this, we construct a serial multiple mediation model (see Figure 1) to probe the influence of school assets on bullying and mechanisms with consideration of empirical findings and relevant theories, and hypothesize that: (1) school assets negatively predict adolescent bullying; (2) ISR will mediate the relationship between school assets and bullying; (3) IGD will mediate the relationship between school assets and bullying; and (4) ISR and IGD play a serial mediating role in the relationship between school assets and bullying.

## Materials and methods

### Participants and procedures

Using a randomized cluster sampling method, students from three secondary schools in Hubei Province were recruited to participate in the two-wave survey 5 months apart. A total of 796 adolescents took part in the first survey, including 428 boys (53.8%) and 368 girls (46.2%), with a mean age of 13.91 years (SD = 2.01). Only participants who completed both questionnaires will be included in the final sample for subsequent analyses, while those with more than 30% missing data will also be excluded. In addition, there were 28 students lost in this study at time 2 for various reasons, such as transfer, absence, and dropping from school, for an attrition rate of 3.52%. This study further compared the differences between the 28 subjects who dropped out at time 2 and the 768 valid subjects using chi-square tests and independent sample t-tests. The results displayed that there were no significant differences between the two groups of subjects in terms of age (χ^2^ = 3.35, *p* = 0.76), gender (χ^2^ = 0.56, *p* = 0.45), grade (χ^2^ = 3.44, *p* = 0.49), family economic status (χ^2^ = 0.51, *p* = 0.78), only child (χ^2^ = 0.39, *p* = 0.53), and T1 school assets (*t* = –0.82, *p* = 0.41), indicating that there was no structural attrition of subjects, that is, the attrition data did not have a large impact on the study results.

All procedures involving human participants were approved by the Research Ethics Committee of the College of Education and Sports Sciences, Yangtze University (No. YZU20210620). Prior to formal data collection, verbal informed consent was received from the school directors and students for this study. Trained teachers and research assistants present standardized instructions detailing the purpose and considerations of the study to participants. Participants were asked to complete the questionnaire on their own. We encouraged participants to be honest and independent in their responses, emphasizing the importance of data confidentiality, the procedure’s safety, and the students’ freedom to participate and withdraw. The whole procedure lasted approximately 25 minutes, and the teachers and students were appreciated afterward.

### Measure

#### School assets at time 1

The Chinese version of the Developmental Assets Profile (DAP) was used to measure the extent to which young people perceive safety, discipline, and care from their teachers and school (91). The scale has 10 items (e.g., “I have a school that gives students clear rules”) and each item is scored on a 4-point scale, from “1 = not at all or rarely” to “4 = extremely or almost always”. A higher total score for all items indicates a higher level of school assets owned. In a previous study, the scale demonstrated good reliability and construct validity (32). In this study, the Cronbach’s alpha for this scale at Time 1 was 0.88.

#### Bullying at time 2

The Bullying Questionnaire was used in this study to measure the frequency of traditional bullying of others in the past six months (92). The questionnaire consists of six questions (e.g., “I (we) forcefully asked someone for money or took or damaged something that belonged to him/her”) and is divided into three dimensions (physical bullying, verbal bullying, and relational bullying), each with two questions, and each scored on a 5-point scale, ranging from “1 = never happened” to “5 = several times a week”. The questionnaire has already exhibited good reliability in a prior study (11). In this study, the Cronbach’s alpha for the bullying questionnaire at Time 2 was 0.77.

#### Intentional self-regulation at time 2

The Intentional Self-Regulation Questionnaire for Adolescents was used to investigate adolescents’ levels of ISR (52, 93). The questionnaire comprises nine questions (e.g., “When I realize that something has little chance of success, I stop doing it, even if it is very important to me”) and contains three dimensions: goal selection, goal optimization, and goal compensation. Each question was scored on a 5-point scale (1 = strongly disagree, 5 = strongly agree), with higher scores representing higher levels of ISR. This questionnaire has shown excellent reliability and construct validity among Chinese adolescents in prior research (94). In this study, the Cronbach’s alpha of the questionnaire at Time 2 was 0.86.

#### Internet gaming disorder at time 2

The IGD Questionnaire was used in this study to assess the propensity of adolescents to experience IGD (95, 96). The questionnaire includes 11 questions (e.g., “Have you ever needed extra money from friends or family because you spent too much money on video game devices, software, or games/internet”), each of which is scored on a 3-point scale from 0 (never) to 2 (often). In order to take into account participants who sometimes experienced addiction symptoms, the resulting data was subsequently recoded (0 = never, 0.5 = sometimes, 1 = often) (97). A high overall score for all questions reflects a higher likelihood of IGD. Similarly, earlier studies have illustrated that the questionnaire has good reliability and construct validity (30, 98). In this study, the Cronbach’s alpha for this questionnaire at Time 2 was 0.87.

### Data analysis

To begin with, this study used Epidata 3.1 for data input and then used SPSS 25.0 for initial collation of the collected data, such as attrition analysis and common method bias analyses. Secondly, we conducted descriptive statistics and correlation analyses of the main variables using SPSS 25.0. Thirdly, as Hayes recommended (99), we examined the serial multiple mediation model (Model 6) using the PROCESS macro in SPSS, and the mediation effects were inspected by the bias-corrected percentile bootstrap method (100). In conjunction with previous literature that suggests IGD and bullying may show differences by gender and grade (21, 74), this study included them as control variables in subsequent model checking.

## Results

### Common method biases analyses

As the data in this study was derived from self-reported questionnaires, there may be common method bias. This study used the Harman’s single factor test to examine the resulting data for common method bias (101). The finding revealed that there were seven factors with a characteristic root greater than one, and the interpretation rate of the first common factor was 22.35%, which was less than the critical value of 40%. Therefore, the common method deviation of the data in this study was not serious.

### Descriptive statistics and correlation analyses

Descriptive statistics for the key variables in the current sample are presented in Table 1. As shown in Table 2, there were significant correlations between all key variables (*ps* > 0.001). Specifically, T1 school assets were positively associated with T2 ISR but negatively associated with T2 IGD and T2 bullying. In addition, T2 ISR was negatively correlated with T2 IGD and T2 bullying, and T2 IGD was positively associated with T2 bullying.

**TABLE 1:** Descriptive statistics of key variables.

Variables	Boys		Girls		Total		
	*M*	SD	*M*	SD	*M*	SD	Range
T1 SA	33.75	5.14	33.56	4.73	34.12	4.97	15–40
T2 ISR	33.85	6.31	33.50	5.95	33.69	6.15	9–45
T2 IGD	1.61	2.13	0.84	1.27	1.25	1.74	0–11
T2 Bullying	6.94	2.13	6.39	1.28	6.68	1.81	6–21

SA, School Assets; ISR, Intentional Self-Regulation; IGD, Internet Gaming Disorder.

**TABLE 2:** Skewness, kurtosis, and correlation coefficients of key variables.

	Variables	Skewness	Kurtosis	1	2	3	4
1	T1 SA	–0.76	0.02	1			
2	T2 ISR	–0.14	–0.11	0.38***	1		
3	T2 IGD	2.11	5.77	–0.16***	–0.16***	1	
4	T2 Bullying	4.19	21.17	–0.25***	–0.21***	0.34***	1

SA, School Assets; ISR, Intentional Self-Regulation; IGD, Internet Gaming Disorder. ***p < 0.001.

### The serial multiple mediation model

This study employed Model 6 in PROCESS (99), controlling for the covariates (gender and grade), with T1 school assets as the independent variable, T2 bullying as the dependent variable, T2 ISR and T2 IGD as mediating variables, and bias-corrected bootstrap tests using 5,000 samples were conducted to ascertain the significance of the serial multiple mediating effects at the 95% confidence interval (CI). If the 95% CI does not contain 0, the results are considered valid. In addition, all variables were standardized prior to formally examining the model, in which gender was coded as a dummy variable.

**FIGURE 2:**
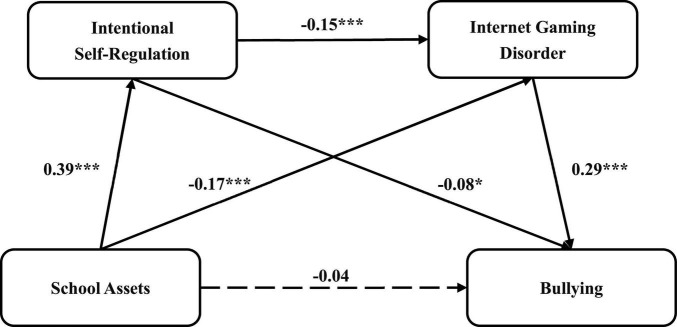
The serial multiple mediation model between school assets and bullying. *p < 0.05, ***p < 0.001.

Figure 2 presents the standardized path coefficients for this serial multiple mediation model. The results of the regression analysis for this model are illustrated in Table 3, suggesting that T1 school assets negatively predicted T2 bullying (*B* = -0.14, *t* = –3.93, *p* < 0.001, 95%CI: [–0.22, –0.07]). However, T1 school assets did not significantly predict T2 bullying (*B* = –0.04, *t* = –1.17, *p* > 0.05, 95%CI: [–0.12, 0.03]) when we inserted two mediating variables, meaning that T2 ISR and T2 IGD completely mediated the effect of T1 school assets on T2 bullying. Moreover, T1 school assets predicted higher levels of T2 ISR (*B* = 0.39, *t* = 11.40, *p* < 0.001, 95%CI: [0.33, 0.46]) and lower levels of T2 IGD (*B* = –0.17, *t* = –4.60, *p* < 0.001, 95%CI: [–0.25, –0.10]). T2 ISR was a negative predictor of both T2 IGD (*B* = –0.15, *t* = –3.98, *p* < 0.001, 95%CI: [–0.22, –0.07]) and T2 bullying (*B* = –0.08, *t* = –2.24, *p* < 0.05, 95%CI: [–0.16, –0.01]). Lastly, T2 IGD positively predicted T2 bullying (*B* = 0.29, *t* = 7.95, *p* < 0.001, 95%CI: [0.22, 0.36]).

**TABLE 3:** Testing the serial multiple mediation effects.

Outcome	Predictors	*R*	*R* ^2^	*F*	*B*	*T*	95%CI
T2 Bullying	Gender	0.21	0.05	12.25***	0.14	3.84***	[0.07, 0.21]
	Grade				0.02	0.55	[–0.05, 0.09]
	T1 SA				–0.14	–3.93***	[–0.22, –0.07]
T2 ISR	Gender	0.39	0.15	44.49***	0.05	1.57	[–0.01, 0.12]
	Grade				0.04	1.25	[–0.03, 0.11]
	T1 SA				0.39	11.40***	[0.33, 0.46]
T2 IGD	Gender	0.35	0.12	26.42***	0.21	6.09***	[0.14, 0.28]
	Grade				0.01	0.28	[–0.06, 0.08]
	T1 SA				–0.17	–4.60***	[–0.25, –0.10]
	T2 ISR				–0.15	–3.98***	[–0.22, –0.07]
T2 Bullying	Gender	0.36	0.13	23.02***	0.08	2.40*	[0.02, 0.15]
	Grade				0.02	0.65	[–0.05, 0.09]
	T1 SA				–0.04	–1.17	[–0.12, 0.03]
	T2 ISR				–0.08	–2.24*	[–0.16, –0.01]
	T2 IGD				0.29	7.95***	[0.22, 0.36]

SA, School Assets; ISR, Intentional Self-Regulation; IGD, Internet Gaming Disorder. Gender was dummy coded such that 1 = boys and 0 = girls. *p < 0.05, ***p < 0.001.

The findings of the mediation effects analysis are given in Table 4. The results revealed that T2 ISR mediated the relationship between T1 school assets and T2 bullying (*B* = –0.033, SE = 0.016, 95%CI: [–0.065, –0.003]). In more detail, school assets predicted a higher level of ISR, which then reduced the occurrence of bullying. Similarly, T1 school assets further mitigated T2 bullying by reducing T2 IGD (*B* = –0.050, SE = 0.017, 95%CI: [–0.087, –0.023]). In terms of chain mediating effects, T1 school assets could increase the level of T2 ISR, thereby diminishing T2 IGD, which in turn further attenuated the likelihood of bullying at time 2 (*B* = –0.017, SE = 0.006, 95%CI: [–0.029, –0.007]). Besides, the effect values of these three indirect pathways accounted for 22.92, 34.72, and 11.81% of the total effect, respectively.

**TABLE 4:** Testing results of all pathways of the serial multiple mediation effects.

Effects	*B*	Boot SE	Boot 95%CI
Total effect	–0.144	0.037	[–0.216, –0.072]
Direct effect	–0.045	0.038	[–0.120, 0.031]
Total indirect effect	–0.099	0.021	[–0.144, –0.061]
Ind 1:T1SA-T2ISR-T2Bullying	–0.033	0.016	[–0.065, –0.003]
Ind 2:T1SA-T2IGD-T2Bullying	–0.050	0.017	[–0.087, –0.023]
Ind 3:T1SA-T2ISR-T2IGD-T2Bullying	–0.017	0.006	[–0.029, –0.007]

SA, School Assets; ISR, Intentional Self-Regulation; IGD, Internet Gaming Disorder.

Bootstrap sample size = 5000.

CI = Confidence Interval.

## Discussion

As the desire for independence and the growing importance of peer relationships among individuals in adolescence continues to expand, this portends great challenges for both parents and educators (30). In view of this, we have adopted a two-wave study with the aim of exploring the relationship between school assets and adolescent bullying. This study tested a multiple mediation model, and the results indicate that school assets have a negative influence on subsequent adolescent bullying, and the association between school assets and bullying was mediated by ISR and IGD. The findings of this study are discussed in detail in the following paragraphs.

First of all, preceding research has concluded that protective factors in the school environment have a significant negative impact on bullying (39, 41, 43, 47). Inspired by the foregoing indirect findings, we explored the direct link between school assets and bullying. The findings demonstrated that school assets predicted eventual teenage bullying in a significant and unfavorable way, confirming hypothesis 1. This finding is consistent with the results of a previous longitudinal study that found developmental assets can significantly reduce subsequent externalizing problem behavior in adolescents (102), which also supports the social-ecological systems theory (34) and the perspective of PYD (36). To put it another way, supportive classroom and school environments are key safeguards that buffer teenagers from bullying and aggression (33, 103, 104). Pellegrini and Long (105) found that youth bullying and aggressive behavior generally increased with the transition to secondary school, and this behavior might be a way of managing peer relationships and dominance as they entered a new social group. In a longitudinal study, Yang et al. (106) observed that adolescents who perceived a better school climate were unlikely to engage in bullying behaviors. Moreover, increased levels of school engagement significantly predicted decreased levels of bullying and aggression in adolescents, making it an essential component of resilience enhancement (107, 108). Also, school insecurity was a strong adverse predictor of bullying (109). This suggests that educators can create a safe and warm school environment by providing quality school assets, which will be efficient in protecting youth from bullying (110).

Second, this study indicated that ISR significantly mediated the relationship between school assets and bullying, supporting Hypothesis 2. This result is in line with the relational-developmental-systems model and the triadic codetermination theory (50, 53), as well as extending further empirical support for the mediating role of ISR in the relationship between external circumstances and developmental outcomes. In particular, ISR arose from beneficial relationships between adolescents and the educational environment (85). Previous research has revealed that Chinese adolescents with more school-type ecological assets have displayed higher levels of ISR (28). The model of motivational dynamics provides a reasonable explanation for this result from the motivational perspective (111). This model states that the school context constantly presents students with goals or demands in various ways and, at the same time, sets new standards for their level of ISR, at which point the motivation to develop ISR is activated. ISR buffered the detrimental impacts of risky behaviors among adolescents, such as aggressive and deviant behaviors (60, 112). It follows that if adolescents are in a mutually enriching relationship with their surroundings, they will feel more connected to the outside world and be less likely to have a tendency to develop problematic behaviors (53). In a prior study, White and Warfa (113) argued that creating a youth-centered ecosystem would be more beneficial in shaping youth into healthy social contributors. Therefore, in promoting positive mental health education, educators need to combine the improvement of school assets with tapping into adolescents’ own initiatives to minimize the occurrence of bullying in schools.

Third, given the previous indirect evidence between these three variables (76, 79), we further examined the mediating role of IGD. As expected, hypothesis 3 was also validated that IGD mediates the relationship between school assets and bullying. In other words, school assets decreased IGD levels, which then in turn reduced bullying. Hence, the present finding expands the literature by suggesting the mediating mechanisms between school assets and bullying and provides theoretical support for social control theory (72). The school is a crucial link in the process of socialization of adolescents from home to society, and enriched school assets are effective in preventing and reducing the risk of problematic behavior among young people. As Benson et al. (35) suggested, adolescents in an ecological context with sufficient developmental resources will have substantial and deep involvement with their surroundings, and they will tend to be less likely to exhibit problematic behaviors and more likely to achieve full development. In addition, IGD was discovered to be a significant predictor of adolescent bullying in our study, similar to previous research (77) and bolstering the GLM (81). As a result, school personnel should concentrate on establishing a comprehensive school asset system, based on which it is necessary to screen students for IGD and bullying in order to offer timely assistance.

Fourth, the present study verified hypothesis 4 by testing the serial mediating impact. The results demonstrated that school assets would indirectly influence bullying through the serial mediation of ISR and IGD. Besides, this result also repeatedly verified the relationship between ISR and IGD, resembling the results obtained in the Chinese context (74, 90). More specifically, better quality school assets facilitate the development of higher levels of ISR among adolescents, which in turn decreases the likelihood of IGD and ultimately reduces the incidence of bullying among peers. The discovery of this serial mediating effect highlights the pivotal role of an individual’s ability to ISR in their surroundings and behavioral outcomes, further supporting SOC theory (86). Earlier research has affirmed the necessity and positive implications of developing self-regulation programs for adolescents (85). Therefore, this indicates that teachers and parents should pay more attention to the development of positive factors in adolescents, as represented by ISR, to improve the ‘goodness of fit’ between the individual and the environment (28).

It is worth noting that this study has the following strengths: First, the sample size for this study was large, with a total of 796 students completing our surveys. Second, this study is based on the PYD perspective and incorporates abundant theories, including social-ecological systems theory, triadic codetermination theory, relational-developmental-systems model, social control theory, GLM, and SOC model, ultimately shedding light on the link between school assets and youth bullying, and revealing the mediating role of ISR and IGD. This will further deepen the previous research and provide empirical support for the above theories. Third, this study has vital practical implications for intervention programs for adolescent bullying. The results of the study confirm that it is important to provide adolescents with school assets, enhance their ISR capacity, as well as pay attention to timely screening for IGD conditions, all of which can contribute effectively to reducing the occurrence of bullying in schools.

Despite the many strengths of this study as described above, there are still some limitations that could be improved. To begin with, all of the data was collected by self-reporting methods, which may have been skewed due to common method bias and social desirability effects. Future research could collect data from numerous sources to improve the accuracy of the data. Second, the data for this study was derived from a sample of cities in Hubei province in China, but there may be considerable differences in the levels of the variables in different regions of the country. In the future, it would be necessary to collect data from a rural sample and further explore these associations to draw more generalized conclusions in the Chinese context. Thirdly, while this study focused solely on the function of the school system in adolescent bullying, other systems can also influence individual development. In order to obtain more integrated results, future studies could incorporate multiple systems such as family, school, and community to probe their association with bullying. Fourth, the results of this study should be generalized with caution. The design of a mediation model constructed from data at only two time points is referred to by Maxwell and Cole as a “half-longitudinal design,” and the potential for significant bias in the results obtained is highlighted (114). Therefore, future longitudinal studies should collect data from three or more waves and analyze the data using structural equation modeling. Lastly, the impact of school assets on various dimensions of bullying was not considered in this study. Xu et al. (109) noted that boys were found to be more involved in physical bullying or verbal bullying, while girls would be more involved in relational and cyberbullying. Subsequent research could further investigate the role of gender factors in the relationship between school assets and different dimensions of bullying, which would further deepen the research on gender disparities in bullying behavior.

### Implications

The findings of this study are also instructive in terms of applications. In the first place, more focus should be placed on the positive aspects of the adolescent development process. The present study indicates that school assets play a crucial role in promoting the behavioral development of youths. Consequently, relevant educators should consider the provision of excellent school assets as a strategy to prevent and reduce the occurrence of bullying in schools among adolescents. For instance, schools can enhance pupils’ school connectedness by improving the physical and cultural settings of the school (54) and schools should also actively carry out practical activities to increase the level of school engagement of students. Furthermore, teachers should also adopt students’ opinions in communication with them to promote their sense of self-esteem and autonomy, which is effective in strengthening teacher-student relationships and creating a positive school climate (55). Secondly, ISR was found to be a key mediator between school assets and youth bullying. This suggests that parents and teachers should spare no effort to help adolescents develop ISR skills so that they are adequately able to cope with the various developmental challenges of puberty. As an example, schools can incorporate ISR development into the regular curriculum of mental health education by combining knowledge and games to equip them with the methods and strategies to effectively facilitate ISR. Thirdly, IGD also has an important role to play in the relationship between school assets and youth bullying. This shows that a conscious focus on teenage IGD will be equally conducive to mitigating bullying to some extent. For example, schools could jointly focus on youth internet gaming usage with parents to offer timely monitoring and intervention.

## Conclusion

The present study explored the relationship between school assets, ISR, IGD, and bullying based on a multiple mediation model. Specifically, adolescents with richer school assets were less likely to engage in bullying behavior. In addition, school assets facilitated an increase in ISR, which further attenuated IGD and ultimately helped to prevent or reduce adolescents from bullying others. These findings suggest that practitioners need to protect adolescents from maladaptive behavior (IGD and bullying) by optimizing their external assets (school assets) and promoting their internal developmental potential (ISR). Overall, positive school assets should be built for adolescents to help them improve their abilities and reduce problematic behavior.

## Data availability statement

The raw data supporting the conclusions of this article will be made available by the authors, without undue reservation.

## Ethics statement

The studies involving human participants were reviewed and approved by the Research Ethics Committee of the College of Education and Sports Sciences, Yangtze University. Written informed consent to participate in this study was provided by the participants’ legal guardian/next of kin.

## Author contributions

XG designed the study. K-NQ collected and analyzed data and drafted the manuscript. G-XX analyzed the data and reviewed and revised the manuscript. XJ and C-SZ participated in data collection. All authors contributed to the article and approved the submitted version.

## Conflict of interest

The authors declare that the research was conducted in the absence of any commercial or financial relationships that could be construed as a potential conflict of interest.

## Publisher’s note

All claims expressed in this article are solely those of the authors and do not necessarily represent those of their affiliated organizations, or those of the publisher, the editors and the reviewers. Any product that may be evaluated in this article, or claim that may be made by its manufacturer, is not guaranteed or endorsed by the publisher.
